# miR-96 and miR-183 differentially regulate neonatal and adult postinfarct neovascularization

**DOI:** 10.1172/jci.insight.134888

**Published:** 2020-07-23

**Authors:** Raphael F.P. Castellan, Milena Vitiello, Martina Vidmar, Steven Johnstone, Dominga Iacobazzi, David Mellis, Benjamin Cathcart, Adrian Thomson, Christiana Ruhrberg, Massimo Caputo, David E. Newby, Gillian A. Gray, Andrew H. Baker, Andrea Caporali, Marco Meloni

**Affiliations:** 1 British Heart Foundation Centre for Cardiovascular Science, Queen’s Medical Research Institute, University of Edinburgh, Edinburgh, United Kingdom.; 2 UCL Institute of Ophthalmology, London, United Kingdom.; 3 Institute of Cardiovascular and Medical Sciences, British Heart Foundation Glasgow Cardiovascular Research Centre, University of Glasgow, Glasgow, United Kingdom.; 4 Bristol Heart Institute, School of Clinical Sciences, University of Bristol, Bristol, United Kingdom.

**Keywords:** Angiogenesis, Vascular Biology, Cardiovascular disease

## Abstract

Following myocardial infarction (MI), the adult heart has minimal regenerative potential. Conversely, the neonatal heart can undergo extensive regeneration, and neovascularization capacity was hypothesized to contribute to this difference. Here, we demonstrate the higher angiogenic potential of neonatal compared with adult mouse cardiac endothelial cells (MCECs) in vitro and use this difference to identify candidate microRNAs (miRs) regulating cardiac angiogenesis after MI. miR expression profiling revealed miR-96 and miR-183 upregulation in adult compared with neonatal MCECs. Their overexpression decreased the angiogenic potential of neonatal MCECs in vitro and prevented scar resolution and neovascularization in neonatal mice after MI. Inversely, their inhibition improved the angiogenic potential of adult MCECs, and miR-96/miR-183–KO mice had increased peri-infarct neovascularization. In silico analyses identified anillin (*ANLN*) as a direct target of miR-96 and miR-183. In agreement, *Anln* expression declined following their overexpression and increased after their inhibition in vitro. Moreover, *ANLN* expression inversely correlated with miR-96 expression and age in cardiac ECs of cardiovascular patients. In vivo, ANLN^+^ vessels were enriched in the peri-infarct area of miR-96/miR-183–KO mice. These findings identify miR-96 and miR-183 as regulators of neovascularization following MI and miR-regulated genes, such as anillin, as potential therapeutic targets for cardiovascular disease.

## Abstract 2

MiR-96 and miR-183 act as molecular switches to regulate endothelial cells angiogenic potential and differentially regulate neovascularization following myocardial infarction in neonatal and adult mice.

## Graphical abstract

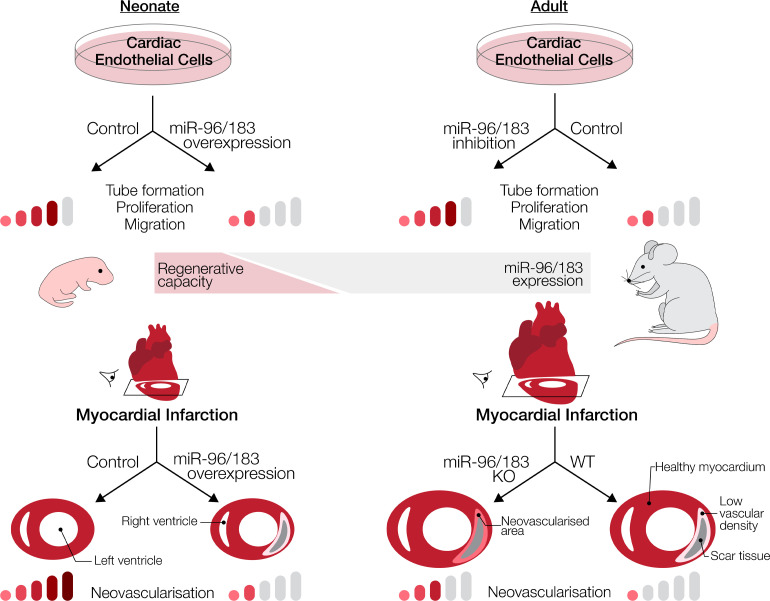

## Introduction

Myocardial infarction (MI) is a major threat to human health and contributes to substantial morbidity and mortality worldwide (1, 2). Conventional treatments aim to promote reoxygenation of the infarcted myocardium, for example, through interventions, such as coronary artery bypass surgery or percutaneous coronary intervention, with the goal of limiting infarct expansion and therefore progression to heart failure (3–5). While a regenerative response is absent in the adult mammalian heart, recent studies have demonstrated that the neonatal mouse and human hearts possess substantial regenerative capacities (6–9). However, this so-called “regenerative window” closes rapidly after birth. Notably, new blood vessel growth by angiogenesis was identified as 1 of the 5 hallmarks of regeneration (10). Thus, the left ventricular (LV) apical resection model of cardiac regeneration in the neonatal mouse heart showed that new blood vessel formation precedes cardiomyocyte repopulation of the regenerating area (11). Accordingly, the neonatal mouse should be useful for identification of key regulators of neovascularization following MI and providing potential therapeutic targets to promote neovascularization and, therefore, cardiac repair following MI in the adult.

MicroRNAs (miRs) regulate many biological processes, including vascular development and angiogenesis (12), and an increasing number of miRs have been shown either to target specific genes involved in angiogenesis or to be modulated by proangiogenic or antiangiogenic stimuli, including miR-221/222, miR-126, and the miR-17/92 cluster (13). Additionally, miR expression is known to be altered in the setting of MI (14), and some miRs, such as miR-590 and miR-199a, induce cardiac repair by promoting cell-cycle reentry of adult cardiomyocytes and inducing their proliferation (15). Furthermore, other miRs, such as those in the miR-15 family, are upregulated in the neonatal mouse heart where they repress several cell-cycle genes and contribute to cell-cycle arrest, thus limiting cardiac regenerative capacity (16). Interestingly, inhibition of the miR-15 family at early stages of life increases cardiomyocytes proliferation and cardiac repair (7). However, the precise role and therapeutic potential of specific miRs in cardiac repair remains poorly investigated. Moreover, miRs are known to act as inhibitory regulators of gene expression by binding to complementary mRNA transcripts (17), and their target genes in the context of neovascularization after MI have not yet been identified.

Here we demonstrate that neonatal cardiac endothelial cells (ECs) have a greater proangiogenic potential than adult cardiac ECs. We further show that neonatal cardiac ECs have a specific miR signature that includes miR-96 and miR-183 as potentially novel therapeutic candidates, and we identify the cytoskeletal regulator anillin (ANLN) as a target of both miR-96 and miR-183. These findings raise the possibility that miR-96/miR-183 and their targets induce a more neonatal-like phenotype in adult cardiac ECs that may be exploited therapeutically to improve postinfarction neovascularization.

## Results

### Neonatal and adult cardiac ECs possess different angiogenic phenotypes.

**Figure 1:**
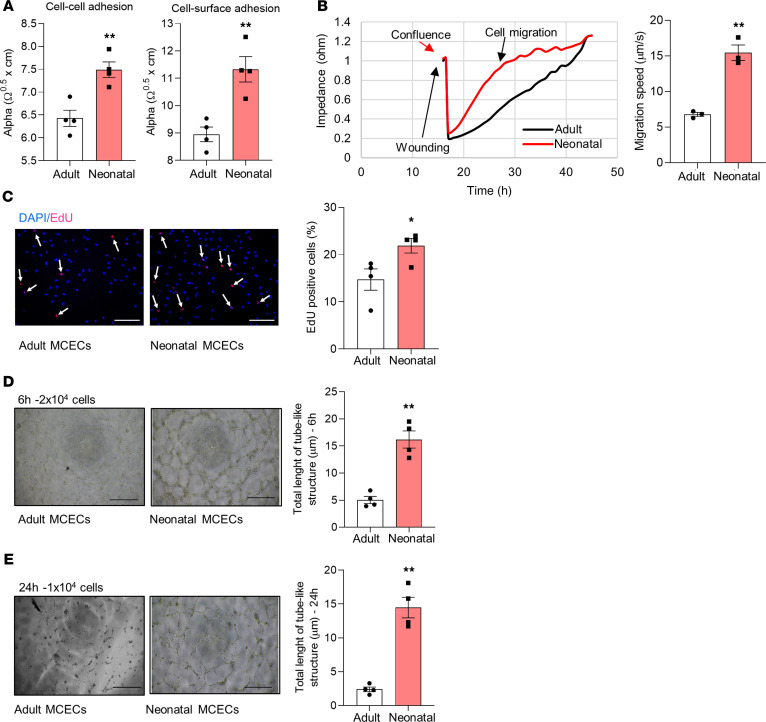
Morphological and functional differences between neonatal and adult MCECs. (**A**) Graphs showing increased cell-cell contacts and cell-surface adhesions of neonatal mouse cardiac endothelial cells (MCECs) compared with adult MCECs. (**B**) Representative impedance curve and quantification showing accelerated migration of neonatal MCECs (red) compared with adult MCECs (black). (**C**). Representative microphotographs and bar graph showing the higher percentage of EdU-proliferating cells in neonatal versus adult MCECs after 24 hours in culture. EdU^+^ cells are shown in red and indicated by arrows, DAPI^+^ nuclei are shown in blue. (**D**) Matrigel experiment with MCECs seeded at high density (2 × 10^4^) showing that adult MCECs are able to form tube-like structures at 6 hours in culture properly and demonstrating that the length of tube-like structures formed by the same number of neonatal MCECs is higher. (**E**) Representative microphotographs and bar graph showing that, whereas 1 × 10^4^ neonatal MCECs form proper tube-like structures at 24 hours, adult MCECs barely form tube-like structure at the same time point. Scale bar: 50 μm. *n* = 3 (**B**); *n* = 4/group (**A**, **C**, **D**, and **E**). *P < 0.05, **P < 0.01 vs. adult MCECs (Student’s *t* test). Data are shown as mean ± SEM.

To assess whether neonatal and adult mouse cardiac ECs (MCECs) have different angiogenic capacities, MCECs from C57BL/6N mice aged P1 (within the regenerative window — herein referred to as neonatal MCECs) and 4 weeks (after the regenerative window — herein referred to as adult MCECs) were used. As shown in Figure 1A, neonatal MCECs had higher cell-cell contact and enhanced cell-surface adhesion in comparison with adult MCECs, as assessed with the electric cell-substrate impedance sensing (ECIS) system. Compared with adult MCECs, neonatal MCECs also had an accelerated migration rate, as evaluated with the ECIS-based automated cell migration assay (Figure 1B), and a higher proliferation rate in culture, measured as 5-ethynyl-2′-deoxyuridine (EdU) incorporation (Figure 1C). When seeded at a high density (2 × 10^4^ cells/cm^2^), both adult and neonatal MCECs form appropriate tube-like structures after 6 hours in culture (Figure 1D). However, neonatal but not adult MCECs formed extensive tube-like structures after 24 hours in culture when seeded at a lower density (1 × 10^4^ cells/cm^2^) (Figure 1E). Taken together, these data indicate that neonatal cardiac ECs possess greater angiogenic potential than adult cardiac ECs.

### The miR signature is different in neonatal and adult MCECs.

**Figure 2:**
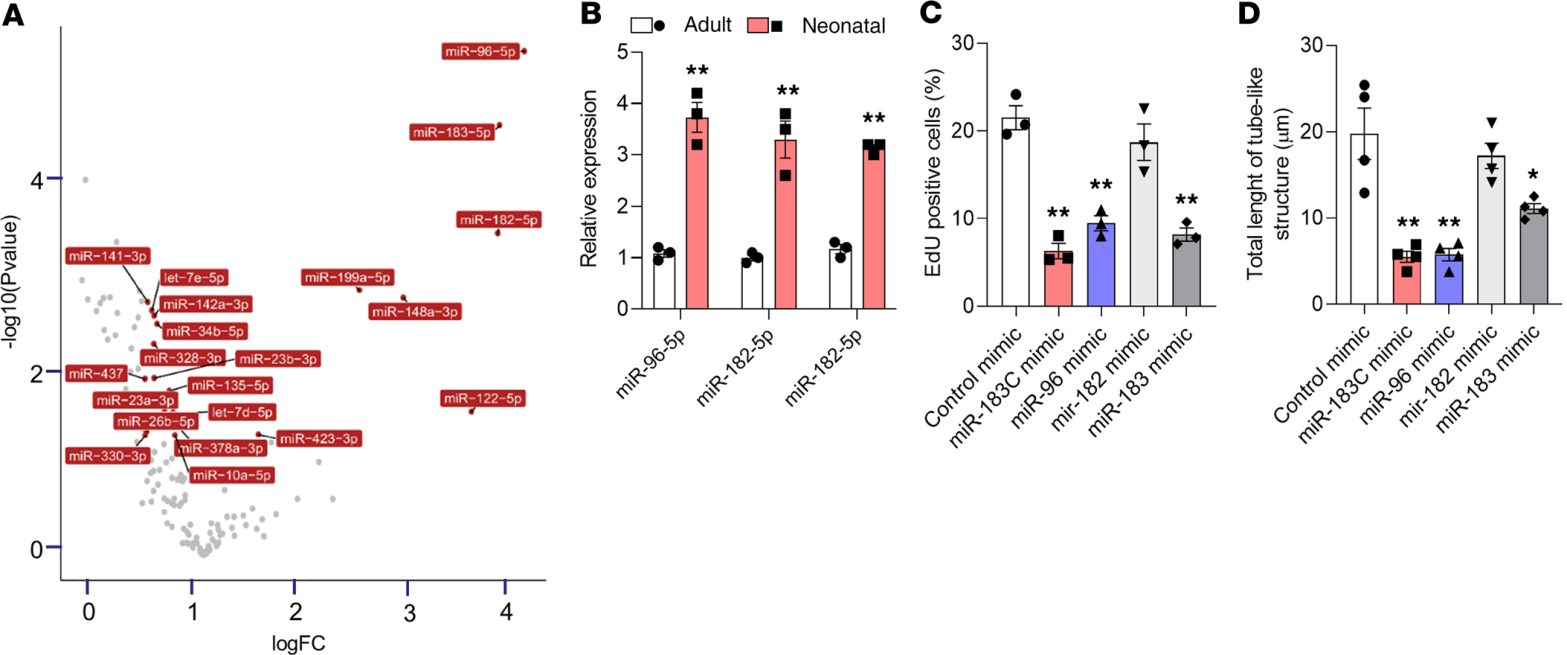
Mimic-mediated overexpression of the miR-183 cluster reduces the angiogenic potential of neonatal MCECs. (**A**) Volcano plot of the significantly differentially regulated miRs in adult mouse cardiac endothelial cells (MCECs) compared with neonatal MCECs. (**B**) Real-time PCR showing upregulation of miR-96-5p, miR-182-5p, and miR-183-5p in cultured adult MCECs compared with neonatal MCECs. Cultured neonatal MCECs were transfected with miR-96, miR-182, or miR-183 (at 25 nM) or with a combination of all mimics of the miR183 cluster (miR-183C - all at 25 nM) or control mimic(s). **P < 0.01 vs. adult (Student’s *t* test). (**C**) Bar graph showing that overexpression of the miR-183 cluster, miR-96 or miR-183, in neonatal MCECs prevents the formation of tube-like structures. (**D**) Bar graph showing that the number of EdU^+^-proliferating cells decreases after overexpression of the miR-183 cluster, miR-96 or miR-183, in neonatal MCECs. *n* = 3/group (**B** and **C**); *n* = 4/group (**D**). *P < 0.05, **P < 0.01 vs. control mimic (1-way ANOVA with Bonferroni’s post hoc test). Data are shown as mean ± SEM.

The greater angiogenic potential of neonatal versus adult MCECs may be the result of several factors. For example, recent findings demonstrate that miRs regulate cardiovascular development, regeneration, and angiogenesis (12, 18). We, therefore, investigated whether a change in miR expression might regulate the angiogenic switch from neonatal to adult cardiac ECs. Expression profiling of 150 miRs in neonatal (P1) and adult (W4) MCECs (Figure 2A) showed that all members of the miR-183 cluster, comprising miR-96-5p, miR-182-5p, and miR-183-5p, were upregulated in adult MCECs compared with neonatal MCECs (Figure 2B). In agreement with this finding, miR-183 and miR-96 are highly enriched in ECs according to the FANTOM5 miRNA Atlas (19). Interestingly, the expression of these miRs in primary MCECs (CD31^+^/CD45^–^) collected within (P1), at the end (P7), and after (P12 and 8 weeks) the regenerative window increased gradually over time (Supplemental Figure 1A; supplemental material available online with this article; https://doi.org/10.1172/jci.insight.134888DS1). These findings raised the possibility that miR-183 and miR-96 are linked to the decreased angiogenic potential of adult MCECs compared with neonatal MCECs.

*Overexpression of miR-96 and/or miR-183reduces the angiogenic capacity of neonatal MCECs in vitro*. To understand whether the miR-183 cluster regulates the angiogenic potential of MCECs we manipulated the expression levels of the miR-183 cluster in neonatal MCECs using synthetic oligonucleotides. While mimic-mediated miR-183 cluster overexpression in neonatal MCECs did not affect their migration rate (Supplemental Figure 2A), it reduced their proliferation rate (Figure 2C and Supplemental Figure 2B) and prevented the formation of capillary-like structures in Matrigel (Figure 2D and Supplemental Figure 2C). Overexpression of the individual members of the cluster showed that miR-96 and miR-183, but not miR-182, reduced the proliferative rate and the formation of the tube-like structure of neonatal MCECs (Figure 2, C and D). These findings suggest that miR-96 and miR-183 regulate the angiogenic potential of MCECs.

### Overexpression of miR-96 and miR-183 impairs angiogenesis and scar resolution following MI in neonatal mice.

**Figure 3:**
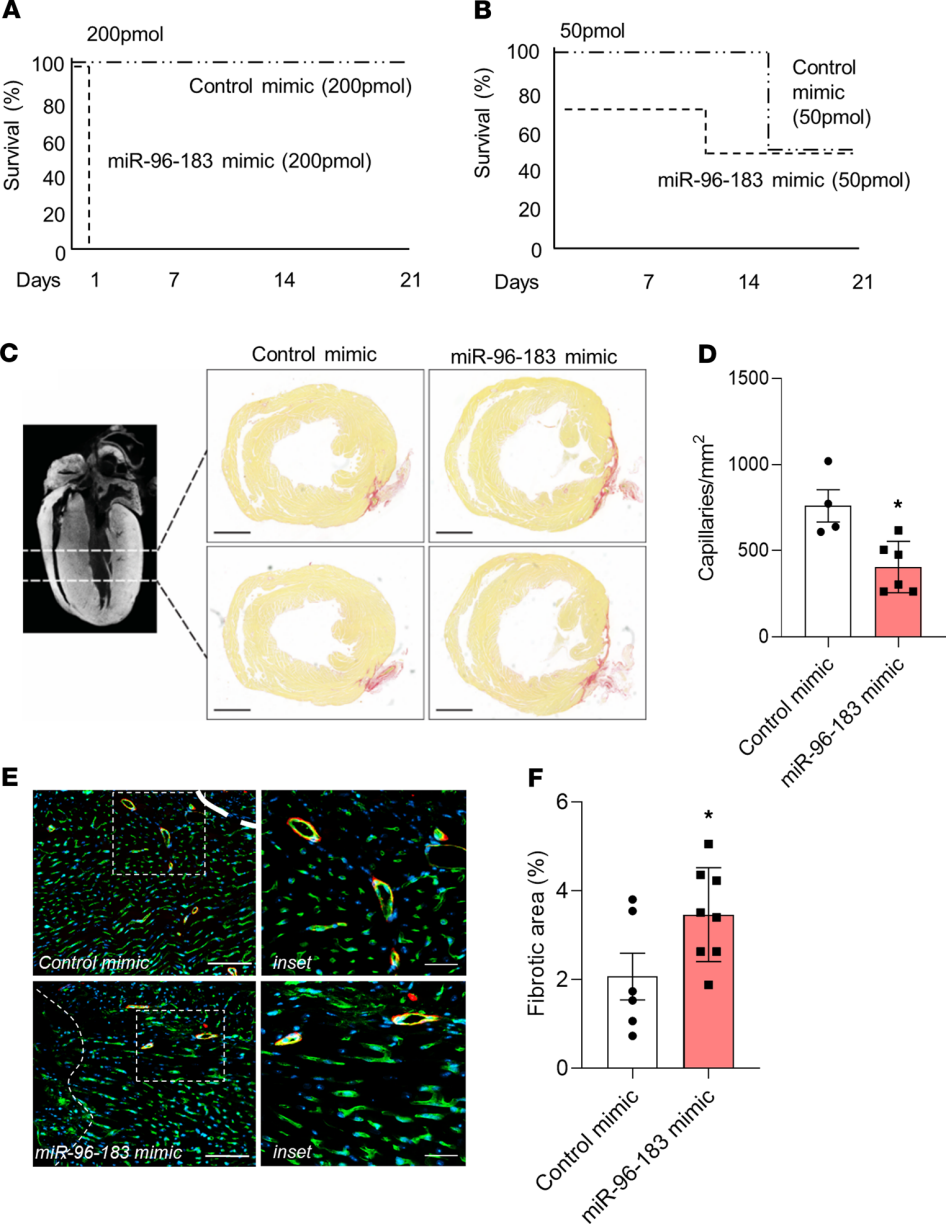
Mimic-mediated overexpression of miR-96 and miR-183 prevents scar tissue resolution and neovascularization of the neonatal mouse heart after MI. (**A** and **B**) Survival curves show that, whereas all neonatal mice injected with a combination of miR-96 and miR-183 mimics at the dose of 200 pmol died within 24 hours after MI and mimic injection (**A**), the mortality rate of mice injected with miR-96 and miR-183 mimics at the dose of 50 pmol was similar to that of control mimic-injected mice (**B**). Representative microphotographs (**C**) and bar graph (**D**) (*n* = 6 control mimic, *n* = 8 miR-96/miR-183 mimic) showing differences in fibrosis deposition (in red, assessed by Picrosirius red staining) between neonatal infarcted hearts injected with either control mimics or miR-96 and miR-183 mimics. Scale bar: 1000 μm. Representative microphotographs (**E**) and bar graph (**F**) (*n* = 4 control mimic, *n* = 6 miR-96/miR-183 mimic) showing the reduced capillary density in the heart of neonatal mice subjected to MI and injected with miR-96 and miR-183 mimics compared with control injected mice. Capillaries are stained with isolectin-B4 (green fluorescence), and arterioles are stained with isolectin-B4 (green fluorescence) and α-smooth muscle actin (red fluorescence). Scale bar: 250 μm (left); 50 μm (right). *P < 0.05 vs. control mimic (Student’s *t* test). Analyses were performed at 21 days after MI. Data are shown as mean ± SEM.

We next used the neonatal mouse model of MI to investigate the effect of miR-96 and miR-183 overexpression in vivo. Following induction of MI, miR-96 and miR-183 mimics were injected into the peri-infarct area. To confirm the feasibility of miR mimic delivery into the neonatal mouse heart after MI, we initially injected a combination of miR-96 and miR-183 mimics at a total dose of 200 pmol (20). However, the survival of pups at 24 hours was dramatically reduced after miR-96 and miR-183 injection compared with control mimic (Figure 3A). We, therefore, decreased the dose to 50 pmol, which did not compromise survival rates compared with the control group (Figure 3B).

At day 1 following MI, RT-qPCR revealed a significant increase in expression of both miR-96 (Supplemental Figure 3A) and miR-183 (Supplemental Figure 3B) in freshly isolated MCECs between the miR-96/miR-183 and control mimic-injected groups. Expression of miR-183, but not miR-96, was also significantly increased in cardiomyocytes (Supplemental Figure 3). At 3 weeks after MI, mice injected with 50 pmol miR-96/miR-183 had increased retention of scar tissue within the LV wall compared with that in the control group (Figure 3, C and D). Notably, the retention of scar tissue was associated with decreased vascularization around the fibrotic tissue (Figure 3, E and F). Together, these data indicate that overexpression of miR-96 and miR-183 inhibits the angiogenic response and scar resolution in the neonatal mouse heart after MI.

### miR-96 and miR-183 exert their vascular effect by targeting Anillin.

**Figure 4:**
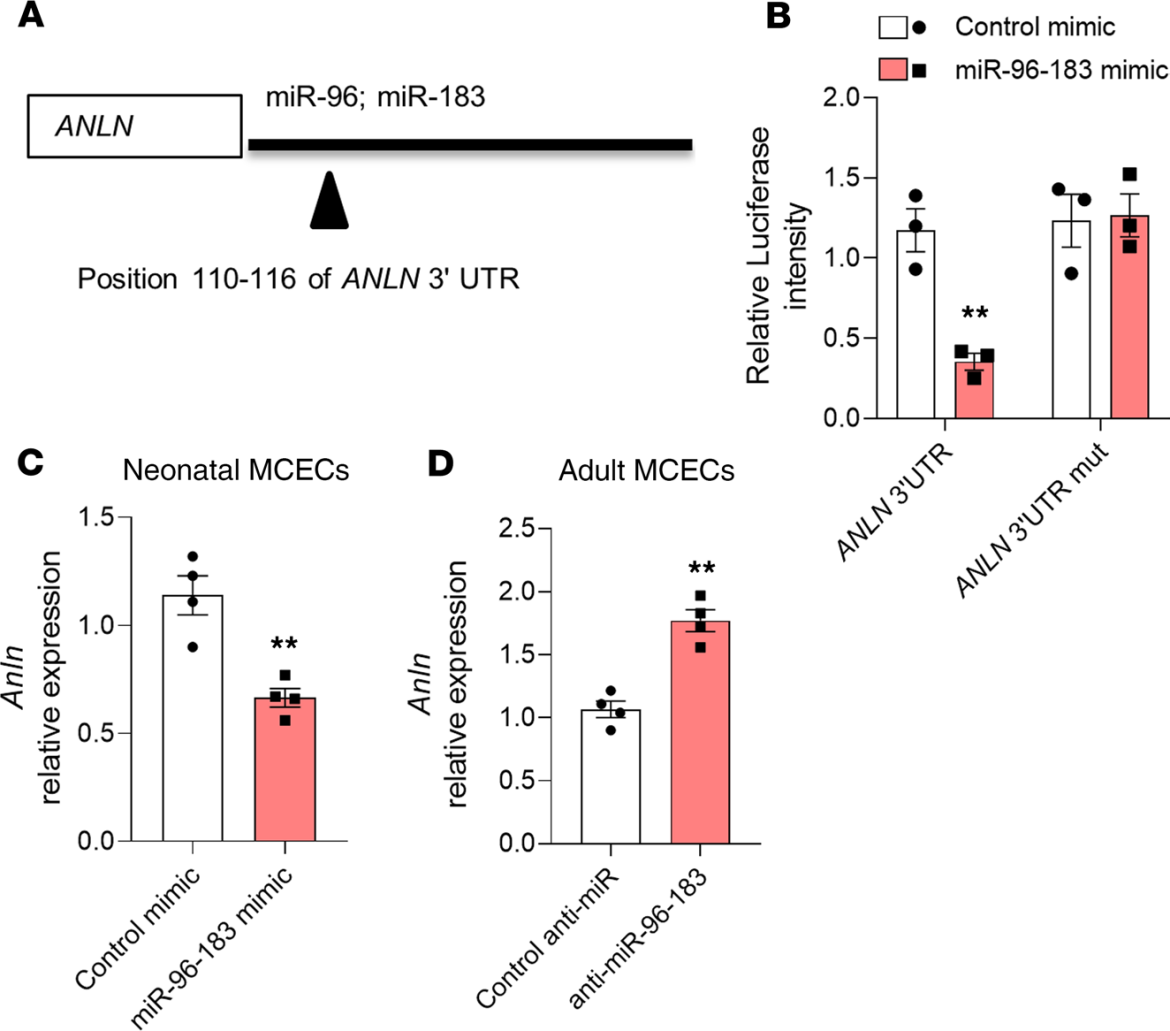
Anillin is a direct target of miR-96 and miR-183. (**A**) Prediction of anillin (ANLN) as a target gene of miR-96 and miR-183 by TargetScan. (**B**) Luciferase assay for *ANLN* 3′-UTR (*n* = 3/group). Luciferase activity at 48 hours after cotransfection of HEK293T cells with both miR-96 and miR-183 or control mimics and *ANLN* 3′-UTR or ANLN 3′-UTR mutated (mut). Relative gene expression of *Anln* after miR-96 and miR-183 overexpression (**C**) or inhibition (**D**) in mouse cardiac endothelial cells (MCECs). (*n* = 4/group). **P < 0.01 vs. control mimic or anti-miR (Student’s *t* test). Data are shown as mean ± SEM.

To identify candidate target genes for repression by miR-96 and miR-183, we performed an in silico analysis of 3′ untranslated (UTR) mRNA and identified genes that are predicted to be targets of both miRs. We identified as common candidate pathways for miR-96 and miR-183 the Hippo pathway, the PI3K/AKT/FOXO signaling pathway, and the regulation of the actin cytoskeleton (Supplemental Figure 4). Among the target genes in this pathway, anillin was predicted as the direct target of both miR-96 and miR-183 by bioinformatics platforms (Figure 4A). A luciferase reporter gene assay in HEK293T cells confirmed that miR-96 and miR-183 bound to the 3′-UTR sequence of *ANLN*, and binding was impaired by a mutation in a predicted binding site of the *ANLN* 3′-UTR (Figure 4B). Consistent with a role for miR-96 and miR-183 in targeting anillin, their overexpression decreased *Anln* mRNA levels in neonatal MCECs (Figure 4C). On the other hand, inhibiting miR-96 and miR-183 in adult MCECs increased the expression of *Anln* (Figure 4D). Together, these findings identify that miR-96 and miR-183 regulate several proangiogenic pathways and identify anillin as one of the direct targets of miR-96 and miR-183.

### Inhibiting the miR-183 cluster improves the angiogenic capacity of adult MCECs.

**Figure 5:**
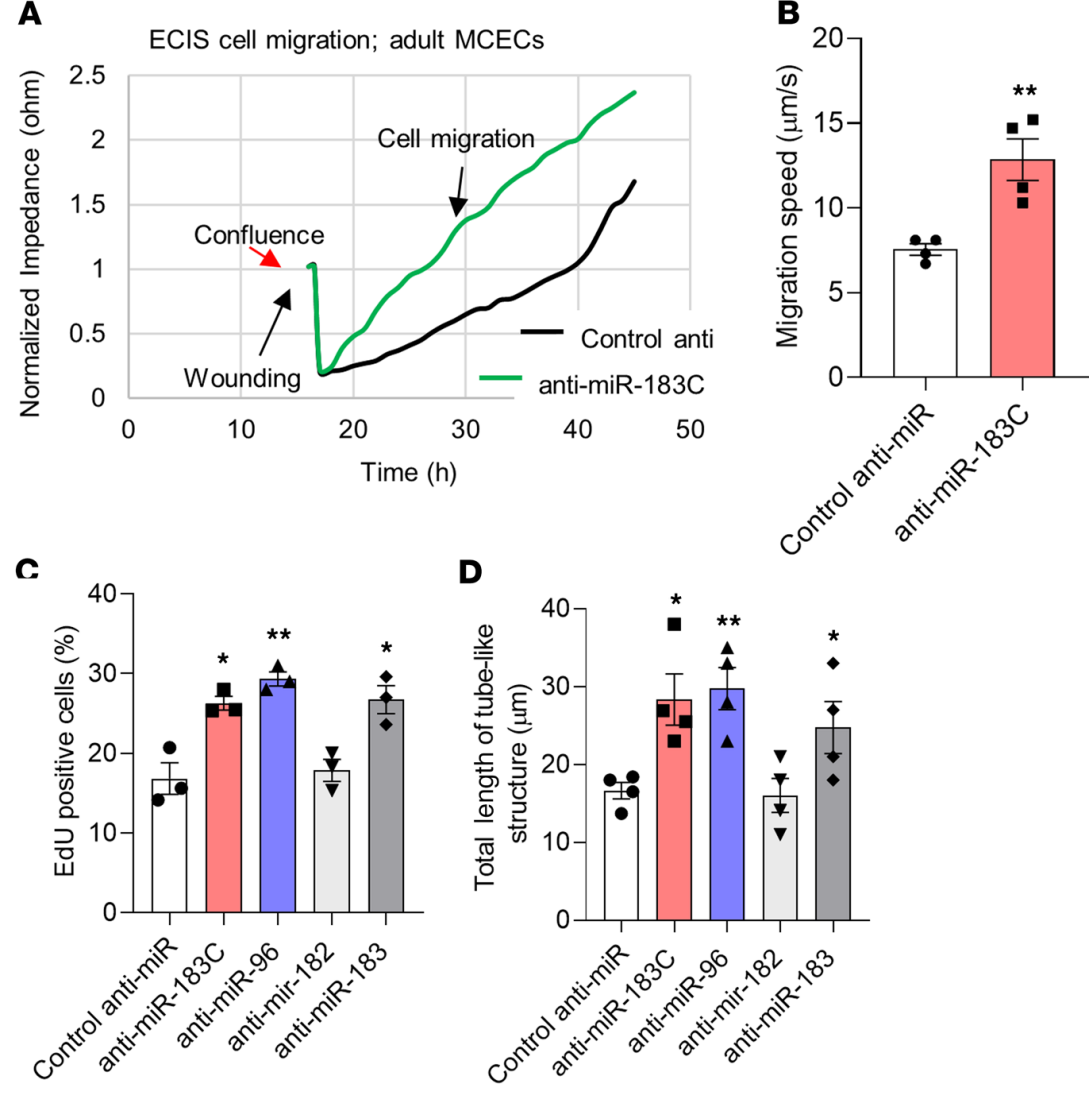
Inhibition of the miR-183 cluster improves the angiogenic potential of adult MCECs. (**A**) Graph showing that inhibition of the miR-183 cluster improves migration of adult mouse cardiac endothelial cells (MCECs, green line) compared with adult MCECs transfected with control mimic (black line). (**B**) Bar graph showing increased migration rate of adult MCECs after miR-183 cluster inhibition. (**C**) Bar graph showing increased tube-like structures formation in adult MCECs transfected with miR-183 cluster or miR-96 or miR-183 inhibitors. (**D**) Bar graph showing a higher number of EdU^+^ cells (increased proliferation) in adult MCECs after inhibition of the miR-183 cluster or miR-96 or miR-183. *n* = 3/group (**C**); *n* = 4/group (**B** and **D**). *P < 0.05, **P < 0.01 vs. control anti-miR (Student’s *t* test or 1-way ANOVA with Bonferroni’s post hoc test. Data are shown as mean ± SEM.

Since overexpression of the miR-183 cluster was found to impair the angiogenic properties of neonatal MCECs, we asked whether inhibition of the miR-183 cluster on adult MCECs may have the reciprocal effect. Inhibiting all members of the miR-183 cluster in adult MCECs improved their rates of migration (Figure 5, A and B), proliferation (Figure 5C and Supplemental Figure 5A), and their ability to form capillary-like structures (Figure 5D and Supplemental Figure 5B). Moreover, inhibiting miR-96 and miR-183 improved adult MCEC proliferation and tube-like formation, whereas miR-182 inhibition did not affect these measures (Figure 5, C and D). Together, these findings confirm that miR-96 and miR-183 inhibit the angiogenic potential of MCECs and suggest that their expression in adult MCECs impairs their regenerative capacity.

### miR-96 and miR-183 inhibition induces neovascularization in the adult mouse after MI.

To investigate whether miR-96 and miR-183 impair angiogenic properties of adult MCECs after MI, we induced MI by permanent occlusion of the left anterior descending (LAD) coronary artery in adult miR-96/miR-183 global KO mice and WT littermates.

**Figure 6:**
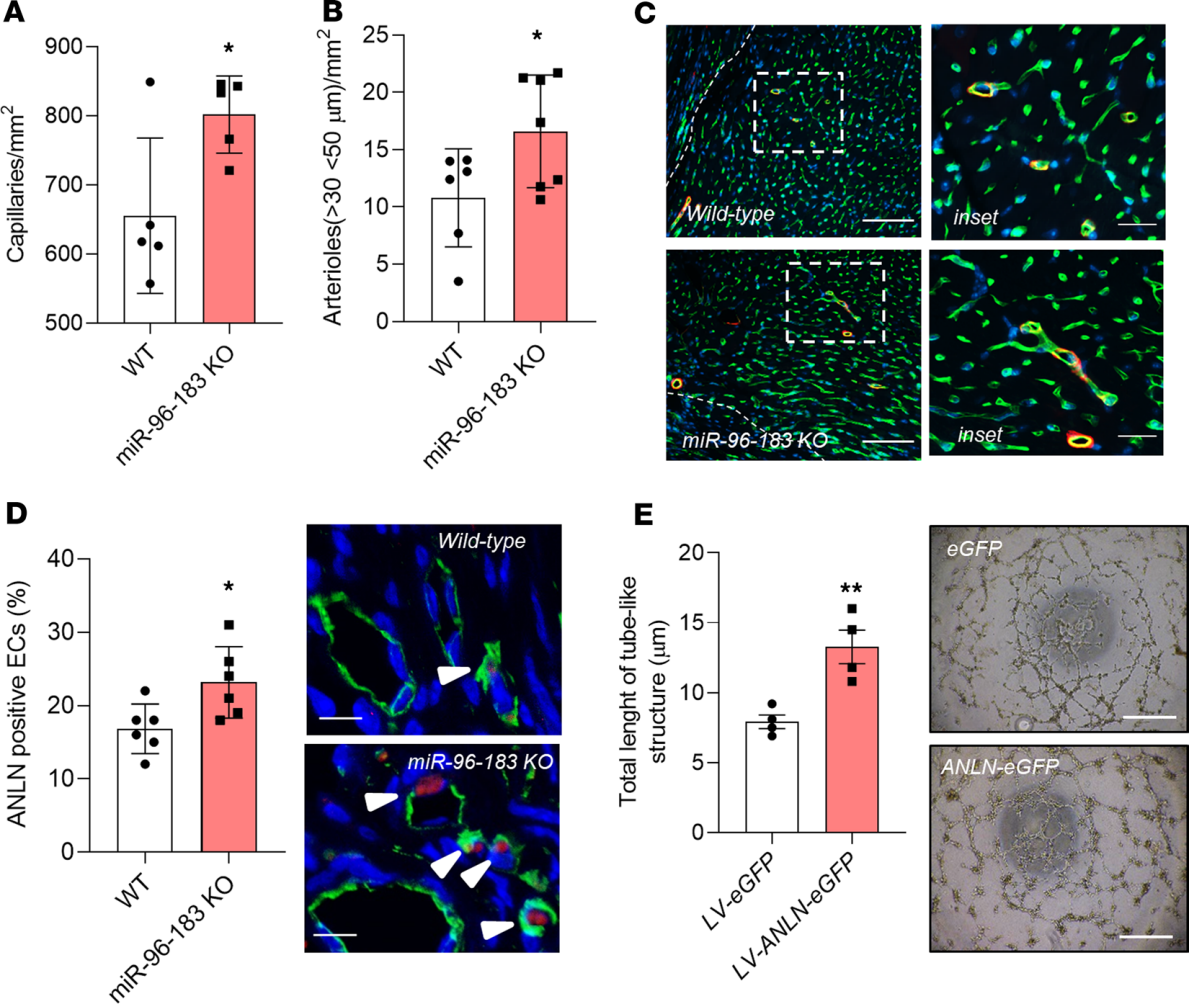
Inhibition of miR-96 and miR-183 increase neovascularization in the adult mouse after MI. (**A**) Bar graph showing the increased number of capillaries (*n* = 5/group) and (**B**) small arterioles (*n* = 6 WT, *n* = 7 miR-96/miR-183 KO) in the peri-infarct of miR-96/miR-183–KO mice at 14 days after MI. *P < 0.05 vs. WT (Student’s *t* test). (**C**) Representative microphotographs showing capillaries (stained by isolectin-B4, green fluorescence) and arterioles (stained by isolectin-B4, green fluorescence, and α-smooth muscle actin, red fluorescence). Scale bar: 250 μm (left); 50 μm (right). (**D**) Bar graph and representative microphotographs showing the increased percentage of ANLN^+^ vessels in the peri-infarct of miR-96/miR-183–KO mice at 14 days after MI (*n* = 6/group). ECs are stained by isolectin-B4 (green fluorescence). ANLN, red fluorescence. Scale bar: 20 μm. *P < 0.05 vs. WT (Student’s *t* test). (**E**) Adult mouse cardiac endothelial cells (MCECs) were transduced with lentiviral vectors *LV-EGFP* or *LV-ANLN-EGFP* and seeded on Matrigel. Representative Matrigel assay images and quantification as total tubule length (*n* = 5/group). Scale bar: 100 μm. **P < 0.01 vs. *LV-EGFP* (Student’s *t* test). Data are shown as mean ± SEM.

While cardiac function and fibrosis content were unchanged (Supplemental Table 1), both capillary and arteriole densities were increased in the peri-infarct of miR-96/miR-183–KO mice compared with those in WT controls (Figure 6, A–C) at 2 weeks after MI.

Notably, the peri-infarct area of miR-96/miR-183–KO mice showed an increased number of vessels expressing the miR-96/miR-183 target ANLN when compared with that of WT mice (Figure 6D). In addition, *Anln* overexpression in adult MCECs in vitro increases the formation of capillary-like structures (Figure 6E). This finding confirms that miR-96 and miR-183 could control the expression of proangiogenic target genes in MCECs during MI.

### The miR-183 cluster in human cardiac ECs.

**Figure 7:**
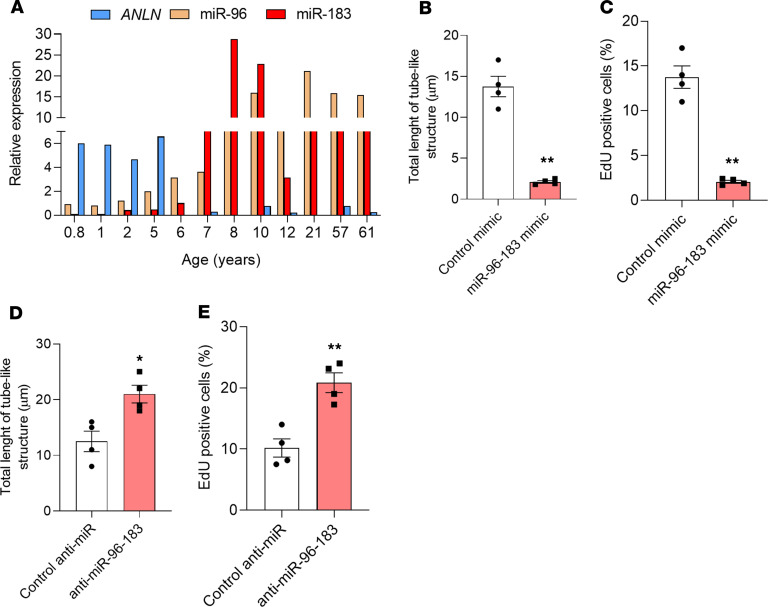
Effect of miR-183 cluster manipulation on HCECs. (**A**) Real-time qPCR showing an age-dependent increased expression of miR-96 and miR-183 and decreased expression of *ANLN* in human cardiac endothelial cells (HCECs) obtained from cardiac biopsies of participants. Commercially available human cardiac microvascular endothelial cells (HCMECs) from adult participants were used to test the effect of miR-183 cluster manipulation in human cardiac ECs. (**B**) Bar graph showing that overexpression of miR-96 and miR-183 prevents the formation of capillary-like structures and (**C**) reduces the percentage of EdU^+^ cells in HCMECs. On the other hand, inhibition of miR-96 and miR-183 increases the length of tube-like structures (**D**) and promotes proliferation (**E**) of cultured HCMECs. *n* = 4/group. *P < 0.05, **P < 0.01 vs. control (Student’s *t* test). Data are shown as mean ± SEM.

To determine whether the miR-183 cluster regulates gene expression in human cardiac ECs (HCECs), we isolated CD31^+^CD45^–^ cells from cardiac biopsies of participants aged <1 year up to 61 years of age (Supplemental Table 2 and Supplemental Figure 6, A–D). While the expression of both miRs was low in HCECs from neonates and infants up to 1 year of age, it increased in HCECs from 8- to 10-year-old children and remained elevated in HCECs isolated from adults (Figure 7A). In contrast, *ANLN* expression was higher in HCECs in children of up to 5 years of age and decreased with age (Figure 7A). Correlation analysis showed a significant inverse correlation between levels of *ANLN* and miR-96 and the age of the patients (Pearson coefficient r = –0.6311, *P* = 0.0278). The expression of both miR-96 and miR-183, therefore, increased with age in primary HCECs, whereas the expression of their target *ANLN* decreased.

Next, we investigated whether manipulation of miR-96 and miR-183 could also affect the regenerative potential of HCECs. For this experiment, we obtained commercial HCECs from adult donors, termed human cardiac microvascular ECs (HCMECs) (see Methods). Whereas mimic-mediated overexpression of miR-96 and miR-183 in these cells reduced the formation of capillary-like structures (Figure 7B and Supplemental Figure 7A) and the proliferation rate (Figure 7C and Supplemental Figure 7B), dual miR-96 and miR-183 inhibition increased both parameters (Figure 7, D and E, and Supplemental Figure 7, C and D).

Together, these findings agree with those obtained for MCECs and suggest that the miR-96 and miR-183 pathway is conserved between mice and humans. Manipulation of these miRs may therefore be a suitable therapeutic target to improve the angiogenic potential of cardiac ECs in humans after MI, although this will likely be in combination with other therapeutic approaches that modulate inflammation and stimulate cardiomyocyte regeneration.

## Discussion

While it has been clear that the angiogenic mechanisms occurring during embryonic and adult life are different (21), the cellular and molecular pathways controlling this switch are relatively poorly understood. Embryonic and neonatal ECs possess a specific transcriptional profile (22), and it is becoming increasingly clear that life stage also influences their phenotype, with “younger” ECs generally thought to possess greater angiogenic capacity (23). Here, we first set out to characterize the phenotypic changes between neonatal and adult primary cardiac ECs.

We observed that neonatal MCECs presented with increased proliferation rates and enhanced migratory capacities when compared with adult MCECs, which resulted in higher angiogenic properties.

miRs are inhibitory regulators of gene expression, which are implicated in the regulation of vascular development (24) and postnatal angiogenesis (25). An increasing number of miRs have been shown to target specific genes involved in angiogenesis or to be modulated by either proangiogenic or antiangiogenic stimuli (12). We, therefore, investigated whether the phenotypical switch between neonatal and adult MCECs correlates with changes in the expression of miRs. Here, we have identified the miR-183 cluster as a regulator of this phenotypical switch. Indeed, overexpression of those miRs in neonatal MCECs decreased their angiogenic capacities, giving them a phenotype like that of adult MCECs. Importantly, we identified that miR-96 and miR-183 were the cause of this effect, whereas the other member of the family, miR-182, did not seem to influence MCECs phenotype.

Angiogenesis is 1 of the 5 hallmarks of cardiac regeneration in neonatal mice, alongside remuscularization, electromechanical stability, resolution of fibrosis, and immunological balance (10). Indeed, angiogenesis preceded cardiomyocyte repopulation of the regenerating area in an apical resection model of cardiac regeneration (11). Understanding the mechanisms underpinning revascularization of the neonatal heart in the context of cardiac regeneration is therefore critical to the development of new therapies to improve repair after MI and hamper the progression to heart failure. We, therefore, sought to determine whether manipulation of the miR-183 cluster could be used to modify outcome following MI in the neonatal mouse.

Intracardiac injection of miR-96 and miR-183 following induction of MI in neonatal mice resulted in their increased expression in MCECs, which was associated with a reduction of capillary density. Together with our in vitro observations, these results suggest that miR-96 and miR-183 can manipulate the phenotype of MCECs in vivo. A failure of the neonatal heart to resorb scar tissue was also observed. While this may be secondary to the reduced capillary density, we cannot rule out that it may be resulting from miR-96 and miR-183 overexpression in other cell types, e.g., cardiomyocytes. Hence, given the upregulation of miR-96/miR-183 in both ECs and cardiomyocytes after delivery, their specific relevance in the observed phenotype cannot be distinguished. Moreover, both EC and cardiomyocyte miR-96/miR-183 expression may well have a role in each cell type and in mediating a crosstalk between these cell types.

To investigate the therapeutic potential of miR-96 and miR-183 further, we performed converse in vitro and in vivo experiments in adult mice. We observed that inhibiting both miRs improved the angiogenic capacities of adult MCECs in vitro, indicating that these miRs may play central roles in regulating the phenotypical switch between neonatal and adult MCECs. These observations were also recapitulated in HCECs, highlighting the translatability of this pathway. Of therapeutic importance, we were able to show that a global KO of the miR-183 cluster was associated with increased vascularization of the peri-infarct zone following MI. However, this was not associated with improved function in our 2-week study. In line with this, there is a disconnect and delay between improved neovascularization and the subsequent improvement in cardiac function and scar formation, which happens later, in patients following MI (26).

To understand the full potential of miR-183 cluster inhibition as a therapeutic target, future studies should look past 2 weeks after MI and focus on the progression to heart failure.

It has been proposed that stimulating angiogenesis may benefit cardiac repair (27). However, our observations show that stimulating angiogenesis is not sufficient to provide the desired functional improvement of cardiac function, likely because other processes would have to be costimulated, including cardiomyocyte proliferation and maturation as well as a beneficial inflammatory environment.

Successful characterization of miR activity relies on identifying the best miR targets matching the observed phenotype and disease (28). Using a bioinformatic approach and an in vitro and in vivo validation, we have identified anillin as the target of miR-96 and miR-183. Anillin is an actin-binding protein that is highly conserved across multiple species and associated with the cell cycle and, specifically, the completion of cytokinesis (29). To analyze the role of cytokinesis during cardiac development and MI, Hesse et al. generated an ANLN-EGFP reporter mouse for the investigation of ANLN in the cell-cycle dynamics in the heart (30), showing nuclear localization of ANLN-EGFP during embryonic heart development. Anillin was found to act as a mitotic marker in pluripotent stem cells and, importantly, marked endoreduplication in myocardial injury (30). Moreover, anillin is also a bona fide marker for cardiomyocyte binucleation, enabling unequivocal discernment of such events from cardiomyocyte division in vitro and in vivo (30).

Recently, transgenic mice expressing an ANLN-EGFP construct under the control of the endothelial-specific Flt-1 promoter have been generated for the monitoring of EC proliferation and cytokinesis (31). ANLN-EGFP signals overlap mainly with the presence of Ki67^+^/PECAM^+^ cells during vascular development and show a decline in EC proliferation between E9.5 and E12.5, therefore supporting the importance of anillin in EC proliferation during vessel development but also its possible role in regulating EC sprouting in miR-96/miR-183–KO mice after MI.

In addition, it is conceivable that *Anln* upregulation in miR-96/miR-183–KO mice might therefore also affect arterial smooth muscle cells (SMCs) and thus increase arterial density through its binding to F-actin and nonmuscle myosin (32). Specifically, the molecular interactions between F-actin and nonmuscle myosin II govern the contraction of the cytoskeleton, which modulates cell shape, division, and migration in SMCs during arteriogenesis (33, 34) However, further experiments would be required to investigate this question.

These findings provide valuable insights into the basic biology underpinning angiogenesis in the neonatal infarcted heart and elucidate potentially novel and fundamentally critical molecular mechanisms that govern angiogenesis after MI during cardiac regeneration. Thereby, our findings highlight the importance and functional role of the miR-183 cluster in EC biology in reestablishing a neonatal-like regenerative phenotype in adult cardiac ECs. Moreover, miR-96 and miR-183 represent putative therapeutic targets in MI.

## Methods

### MCECs.

MCECs were either purchased from Cell Biologics or isolated in-house from postnatal C57BL/6N mice. In-house MCECs were obtained from pooled hearts of mixed-sex C57BL/6N mice aged between P1 and 8 weeks. Briefly, hearts were enzymatically digested using a Neonatal Heart Dissociation Kit (30-098-373, Miltenyi Biotec) in combination with the gentleMACS Dissociator (130-093-235, Miltenyi Biotec) to obtain a single-cell suspension of all cardiac cells. Cardiomyocytes and fibroblasts were removed using a 40-μm cell strainer. CD31^+^/CD45^–^ ECs were then obtained using microbeads (CD31 Microbeads, mouse: 130-097-418 Miltenyi Biotec; CD45 Microbeads, mouse: 130-052-301, Miltenyi Biotec) and the Miltenyi Biotec MACS sorting technology according to the manufacturer’s instructions. Immediately after isolation, cells were either cultured in Complete Mouse Endothelial Cell Medium with growth factors supplements (PeloBiotech) with 10% FBS or used from passage 2–5. Commercial MCECs from Cell Biologics (catalog C57–6024) were isolated from 1-day-old (P1) and 4-week-old C57BL6 mice (35, 36). Cells were from pooled hearts of mixed-sex donors, cultured on gelatin-coated plates, and maintained in Complete Mouse Endothelial Cell Medium with growth factors supplements (PeloBiotech) with 5% FBS and used from passage 2–5, according to the manufacturer’s recommendations. MCECs were used for functional assays (as detailed below) and miR PCR arrays.

To analyze the miR-96 and miR-183 expression in MCECs and cardiomyocytes after miR-96/miR-183 injection, the protocol described above was applied; however, cardiomyocytes were collected after ECs depletion and cultured in a 6-well plate with DMEM 10% FBS for 2 hours to allow fibroblasts to attach to the plate. Cardiomyocytes were then collected from the media by centrifugation.

### HCECs.

HCMECs were purchased from PromoCell. HCMECs were isolated from a healthy male participant (49 years of age) and were cultured in EGM-2 (EBM-2 added with growth factors and other supplements, PromoCell) with 10% FBS and used between passage 2 and 4. Additionally, primary CD31^+^/CD45^–^ HCECs were obtained from Massimo Caputo, University of Bristol (REC reference: 15/LO/1064; IRAS ID: 156551). Cardiac biopsies from the right ventricle of patients undergoing surgery with a diagnosis of Tetralogy of Fallot/pulmonary atresia were collected, and HCECs were isolated using the Neonatal Heart Dissociation Kit and MACS technology (Miltenyi Biotec). Immunocytochemistry for the endothelial markers von Willebrand factor and CD31 was performed to characterize HCECs in culture. Briefly, cells were fixed in 4% buffered paraformaldehyde and permeabilized with 0.1% Triton X-100 before incubation with primary antibody for CD31 (1:100; clone JC70A, GA610, Dako) and von Willebrand factor (1:100, GA527, Dako). Alexa Fluor–labeled secondary antibodies (1:1000, Molecular Probes/Invitrogen) were used to detect primary antibodies. Staining with DAPI (1:2000; Vector Laboratories Inc.) was used to identify nuclei. Images were obtained using an Axio Observer.Z1 microscope with Zen Blue software (Zeiss).

### Cell transfection.

MCECs and HCMECs were transfected with 25 nM miR-96, miR-182, and miR-183 miRIDIAN mimics and inhibitors (or controls) (Dharmacon) using Lipofectamine 2000 Reagent (Invitrogen). Individual miR transfections were performed using 25 nM mimics or inhibitors; cotransfection experiments using 2 (miR-96 and miR-183) or 3 (miR-96, miR-182, and miR-183) miRs were performed using the total dose of 25 nM of either mimics or inhibitors.

### ECIS system.

Commercial neonatal and adult MCECs were plated on the ECIS chip array (8W1E or 8W10E) in order to assess cell-cell and cell-surface adhesion properties as well as their migration rate. Adhesion and migration were additionally evaluated in commercial neonatal MCECs transfected with miR-96 and miR-183 mimics (and controls) and commercial adult MCECs after transfection with miR-96 and miR-183 inhibitors (and controls). The migration speed was calculated in μm/h, Rb, and α as reported in Giaever and Keese (37).

### EdU proliferation assay.

Proliferation assay was performed on MCECs and HCMECs transfected with miR mimics, miR inhibitors, or controls using the Click-iT EdU Alexa Fluor 555 imaging kit (Thermo Fisher Scientific). At 48 hours after transfection, ECs (4 × 10^3^ cells/well) were seeded in 96-well plates and incubated with EdU (10 μmol/l) for 24 hours. Cells were fixed with buffered 4% PFA (MilliporeSigma) in PBS for 15 minutes at room temperature and stained following the manufacturer’s instructions. Nuclei were stained with DAPI. Experiments were performed in triplicate. Cells were analyzed at a ×400 magnification, and the percentage of EdU^+^ cells was determined.

### Matrigel assay.

MCECs or HCMECs were seeded in 6-well plates (1.5 × 10^4^ cells/well); transfected with miR mimics, miR inhibitors, or controls for 48 hours; and then trypsinized and plated in a flat-bottomed 96-well plate coated with growth factor–enriched Matrigel (Matrigel Matrix, Basement Membrane, BD Biosciences). On adult MCECs, Matrigel was also performed by seeding the cells at a density of 1 × 10^4^ cells/well. Endothelial network formation was quantified at 6, 12, and 24 hours in randomly captured microscopic fields (magnification ×40) by counting the length of vascular-like structures.

### RNA extraction miR expression profiling and analysis.

Total RNA was extracted using the miReasy kit (QIAGEN). Real-time quantification to measure miRNA was performed with the TaqMan miRNA reverse transcription kit and miRNA assay (Thermo Fisher Scientific). miRNA expression was normalized to the U6 small nucleolar RNA (snRU6). For mRNA analysis, cDNA was produced using the QuantiTect Reverse transcription kit (QIAGEN). Expression was normalized to 18S ribosomal RNA. Real-time qPCR was used to measure the expression of miR-96 (assay ID: 000186; Thermo Fisher Scientific), miR-182 (assay ID: 000597, Thermo Fisher Scientific), miR-183 (assay ID: 002269; Thermo Fisher Scientific), and snRU6 (assay ID: 001973; Thermo Fisher Scientific). *ANLN* and 18S rRNA preoptimized primers were obtained from MilliporeSigma (KiCqStart Primers). miR expression profiling of 150 miRs was performed using mouse miScript miRNA PCR arrays (96-well format; QIAGEN) and SYBR Green–based real-time PCR analysis, using the Roche LightCycler real-time PCR system. Relative expression was calculated using the 2-ΔΔ^Ct^ method (38). The web-based miScript miRNA PCR array data analysis tool was used to analyze the real-time PCR data (QIAGEN).

### Animal work.

C57BL/6N mice bred in-house from stock originally obtained from Harlan and aged between P1 and 10 weeks. miR-96/miR-183–KO mice (Mirc40; EMMA ID EM:10856; international strain designation C57BL/6N-Atm1Brd Mirc40^tm1Hmpr/WtsiOulu^) were obtained from the University of Oulu (Finland) European Mouse Mutant Archive (EMMA).

### Neonatal and adult mouse models of MI.

Female miR-96/miR-183–KO mice and C57BL/6N (controls) aged 8–10 weeks were used for adult MI studies. MI in adult mice was induced as previously described (39). Briefly, anesthetized mice (isoflurane) were orally intubated and artificially ventilated using a Minivent mouse ventilator (Harvard Apparatus). The chest was opened through an incision in the intercostal space and MI was induced by permanent ligation of the proximal LAD coronary artery by using a 7.0 Mersilene suture (Ethicon). The surgical wound was sutured, and animals were allowed to recover.

Both male and female C57BL/6N mice aged P1 were used for neonatal MI studies. MI was induced by ligation of the LAD coronary artery in P1 mice as described in refs. 8, 40. Briefly, anesthesia was induced by a combination of hypothermia and breathable (isoflurane) anesthetic. The animal was secured onto an ice pack for the duration of the procedure to maintain adequate anesthesia. A 5-mm skin incision was placed over the left thorax above the fifth rib, the skin was blunt dissected, and a small opening was created at the fifth intercostal space to visualize the left pulmonary lobes and heart. The LAD was identified and ligated (9-0 Ethilon, Ethicon). Using a 32-gauge insulin syringe, 200 pmol or 50 pmol of either miR mimics or control (total volume of 5 μL) was injected within the LV wall below the ligation. The ribs, pectoral muscles, and skin were closed (8-0 Prolene, Ethicon). Analgesia was administrated with one drop of 1:50 bupivacaine (Marcaine Polyamp Steripack 0.25%) in saline onto the wound. The entire litter was tattooed for identification purposes and returned to the nest.

### In vivo high-resolution ultrasound.

Cardiac functional parameters of adult mice were assessed at baseline (1 day before induction of MI) and at 2 weeks. Briefly, anesthesia was induced using isoflurane in medical O_2_ at concentrations of 2%–3%. Isoflurane was used for anesthesia maintenance at around 2%. Rectal temperature was monitored (36.5°C–38°C) with a Physitemp RET-3 probe. Thoracic hair was removed using commercially available electric shavers (Philips Hairclipper, HC9490/15) and depilatory cream (Veet Hair Removal Cream for sensitive skin). Conductive ultrasound gel (Parkers Lab) was then applied to each paw to record ECG signals. Prewarmed Aquasonic 100 ultrasound transmission gel was applied onto the abdomen avoiding bubble formation. Image acquisition was performed on a Visualsonics Vevo 770 ultrasound biomicroscope. Cardiac function was assessed using standard parasternal long-axis images of the LV in M-mode and ECG-gated kilohertz visualization B-mode. Functional measurements were extracted using Vevo 770 software.

### Histological evaluation in the mouse heart.

Fresh heart samples were fixed in 10% formalin for 24 hours and subsequently placed in 70% ethanol for at least 24 hours before being embedded in paraffin. Paraffin-embedded samples were cut into sequential 4-μm-thick sections on superfrost slides (VWR). Slides were deparaffinized and rehydrated. Histological analysis was performed on Picrosirius red–stained sections. Capillary and arteriole densities were determined using fluorescent microscopy on sections stained with Alexa Fluor 488–conjugated isolectin-B4 (1:100, I2141, Molecular Probes), Cy3-conjugated α-vascular smooth actin (1:200, C6198, MilliporeSigma), and Anillin (1:100, ab154337, Abcam).

### Bioinformatics prediction and pathway analysis of miR-183 cluster target genes.

Computational prediction of miR-183 cluster target genes was done using a published algorithm (TargetScan 7.2; http://www.targetscan.org). MirPath (http://snf-515788.vm.okeanos.grnet.gr/) was used to perform miRNA pathway analysis.

### Luciferase assay.

Luciferase assay has been performed as previously described (41). *ANLN* 3′-UTR vector was from the LightSwitch 3′-UTR Reporter GoClone Collection (Active Motif). Primers for 3′-UTR mutation at the position 110–116 are as follows: forward 5′-CGAAAGGGTTTaataattTATTCACTACGTA -3′ and reverse 5′- TACGTAGTGAATAaattattAAACCCTTTCG -3′, where lowercase letters represent the miR-96 and miR-183 mutated seed sequence. Mutation was performed using GeneArt mutagenesis system (Thermo Fisher Scientific). Luciferase constructs were transfected into HEK293T cells together with miR-96 and miR-183 mimics or p-SV-β-gal control vector. Cells were cultured for 48 hours and assayed with the Luciferase and β-Galactosidase Reporter Assay Systems (Promega). Luciferase values were normalized to protein concentration and β-galactosidase activity.

### Lentiviral vectors.

Lentiviral vectors pLV: *ANLN-EGFP* and control pLV: *EGFP* were purchased from Cyagen. Production and purification of lentiviral particles were performed at Edinburgh University Core facilities. Cardiac ECs were transduced with 10 MOI of lentiviral particles in full media containing 8 μg/mL polybrene overnight. Transduced cells were FACS sorted to have a selected population expressing ANLN-EGFP and EGFP as control.

### Statistics.

Statistical analysis was performed using GraphPad Prism 5 software. Data are expressed as mean ± SEM. Student’s unpaired 2-tailed *t* test was used for comparison of 2 groups. For comparison among more than 2 groups, a 1- or 2-way ANOVA with post hoc Bonferroni’s multiple comparison test was used. A *P* value of less than 0.05 was interpreted to denote statistical significance.

### Study approval.

All animal experiments were approved by and performed in accordance with the University of Edinburgh Animal Welfare and Ethical Review Body and the UK Home Office. Experimental procedures involving human participants were performed in accordance with the Declaration of Helsinki and were approved by the responsible ethics committees (NHS Health Research Authority, Whitefriars, Lewins Mead, Bristol, United Kingdom). Research on clinical samples was performed in agreement with the Human Tissue Act. Written informed consent was received from participants before inclusion in the study (REC reference: 15/LO/1064; IRAS ID: 156551).

## Author contributions

AC and MM developed and designed the research study. RFPC, M. Vitiello, M. Vidmar, SJ, DI, DM, BC, AT, AC, and MM conducted the experiments and acquired data. MC provided human samples. CR, DEN, GAG, and AHB edited the manuscript. RFPC, AC, and MM wrote and edited the manuscript, with contributions from all the authors.

## Supplementary Material

Supplemental data

## Version 1 06/16/2020

In-Press Preview

## Version 2 07/23/2020

Electronic publication
