# Supplementary material for: miR-96 and miR-183 differentially regulate neonatal and adult postinfarct neovascularization
Source: JCI Insight. 2020 Jul 23;5(14):e134888. doi: 10.1172/jci.insight.134888 (PMC7453899; doi:10.1172/jci.insight.134888)
Supplement: Supplemental data [file jciinsight-5-134888-s041.pdf]

Supplemental Material

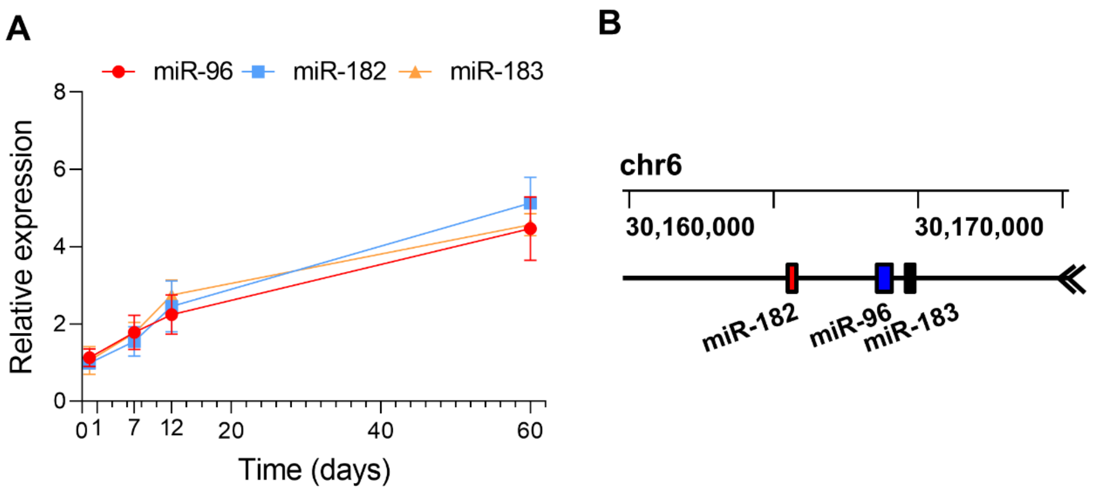

Supplementary Figure 1. MiR expression profiling in neonatal and adult MCECs. (A) The expression of all members of the miR-183 cluster gradually increases in MCECs at different time points across the regenerative window (P1, P7, P12 and 8 weeks). (B) Genomic structure of the miR-183 cluster in the mouse.

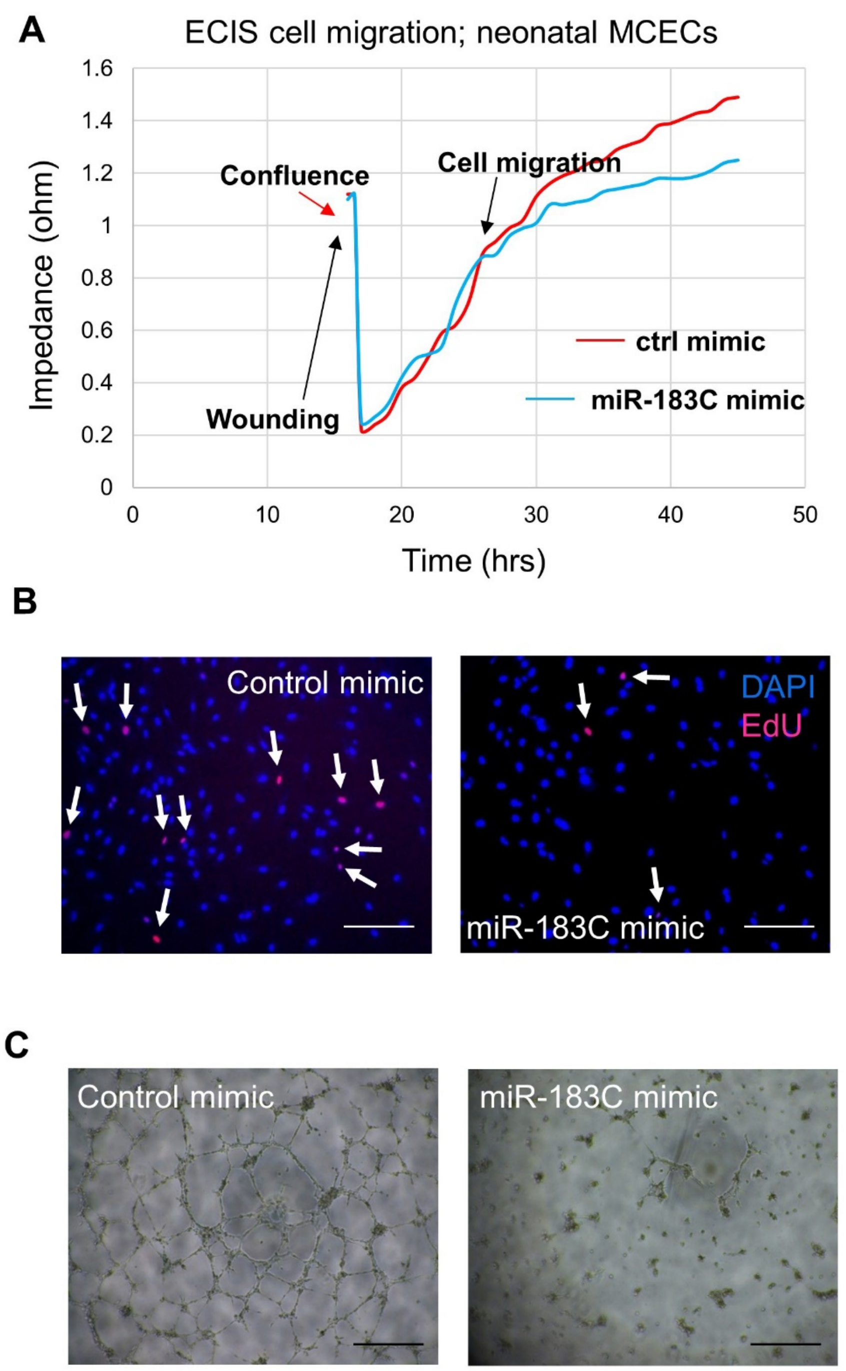

Supplementary Figure 2. Effects of miR-183 cluster overexpression in neonatal MCECs. (A) Graphs show that mimic-mediated overexpression of the miR-183 cluster does not affect migration of neonatal MCECs (red line) compared to neonatal MCECs transfected with control mimic (blue line). (B) Representative microphotographs showing that the number of EdU+ proliferating cells decreases after overexpression of the miR-183 cluster in neonatal MCECs. EdU^+ cells are shown in red and indicated by arrows, $DAPI^+$ nuclei in blue. Scale bar = 50μm. (C) Representative microphotographs showing that overexpression of the miR-183 cluster in neonatal MCECs prevents the formation of tube-like structures. Scale bar = 50μm.

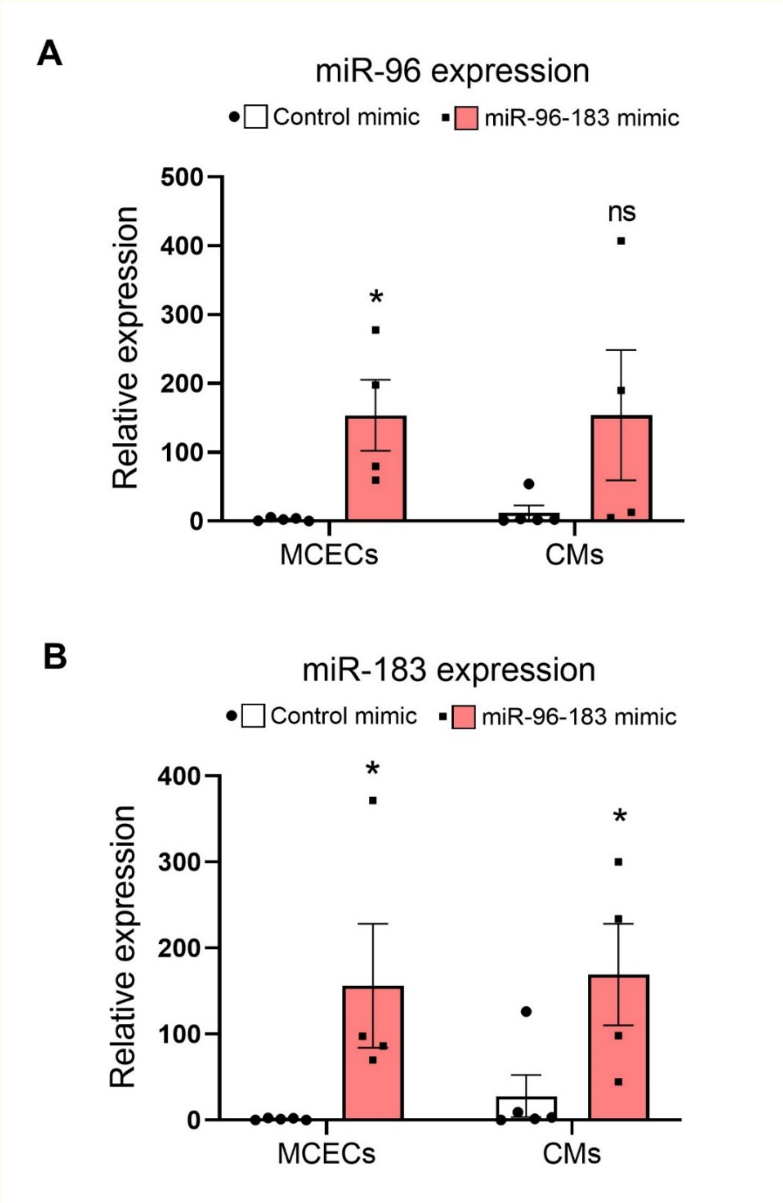

Supplementary Figure 3. miR-96 and miR-183 mimics intra-myocardial injections increases their respective expression levels in MCECs and cardiomyocytes. (A) Real-time PCR showing expression levels of miR-96 at day 1 following MI in freshly isolated MCECs and cardiomyocytes (CMs). (B) Real-time PCR showing expression levels of miR-183 at day 1 following MI in freshly isolated MCECs and CMs. n = 5 for control mimic and n = 4 for miR96 and miR183 injected groups. *p < 0.05 vs control anti-miR. (Student's *t*-test). Data are expressed as mean ± SEM.

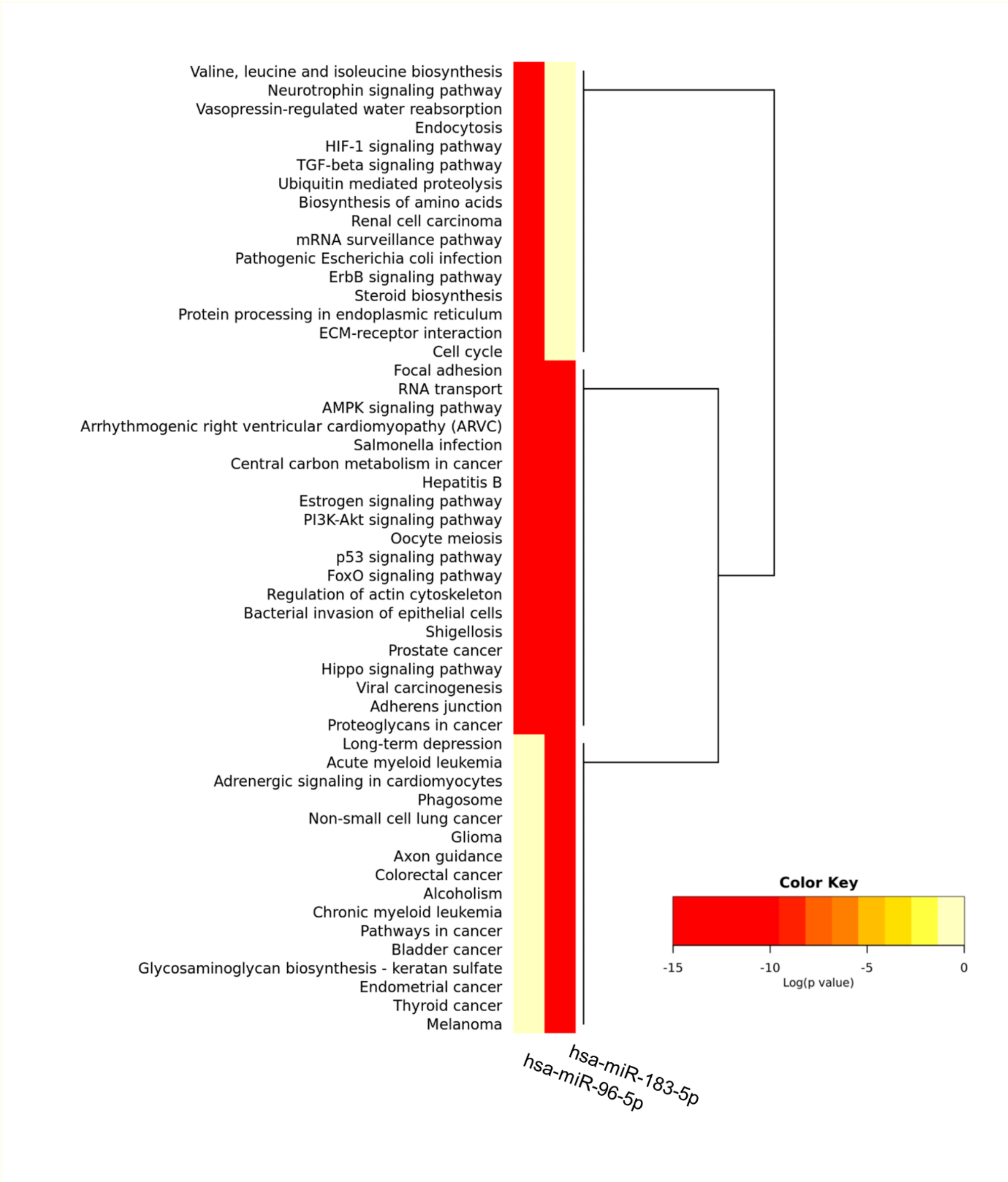

Supplementary Figure 4. MiR pathway analysis. miR pathway analysis was performed using DIANA-mirPath webserver. Target genes were identified by Tarbase and allocated to KEGG pathways. Common pathways for miR-96 and miR-183 are shown in red.

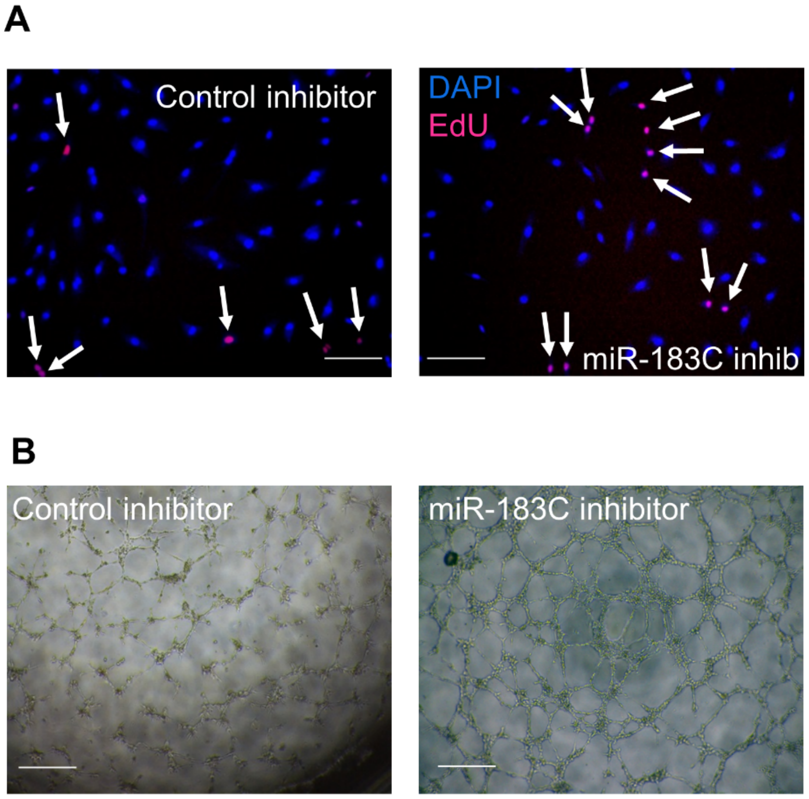

Supplementary Figure 5. Effects of miR-183 cluster inhibition in adult MCECs. (A) Representative microphotographs showing the increased number of EdU+ proliferating cells after inhibition of the miR-183 cluster in adult MCECs. EdU^+ cells are shown in red and indicated by arrows, $DAPI^+$ nuclei in blue. Scale bar = 50µm. (B) Representative microphotographs showing that inhibition of the miR-183 cluster in adult MCECs induces the formation of tube-like structures. Scale bar = 50µm.

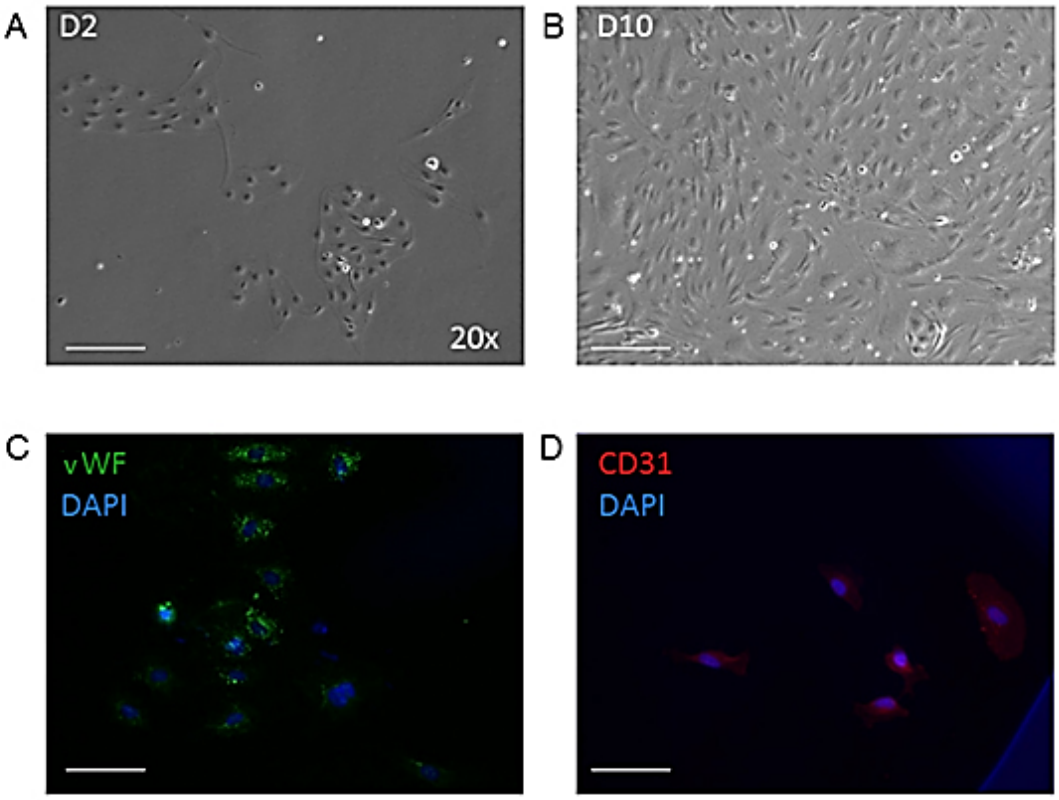

Supplementary Figure 6. Isolation, culture and characterisation of human cardiac ECs. Human cardiac ECs (HCECs) were isolated from the heart of neonatal subjects in collaboration with Prof. Massimo Caputo, University of Bristol. HCECs at 2 days (A) and 10 days (B) of culture. Characterisation of HCECs in culture using the endothelial markers Von Willebrand Factor (vWF, in green; C) and CD31 (in red, D). $DAPI^{+}$ nuclei are depicted in blue. Scale bar = 50μm

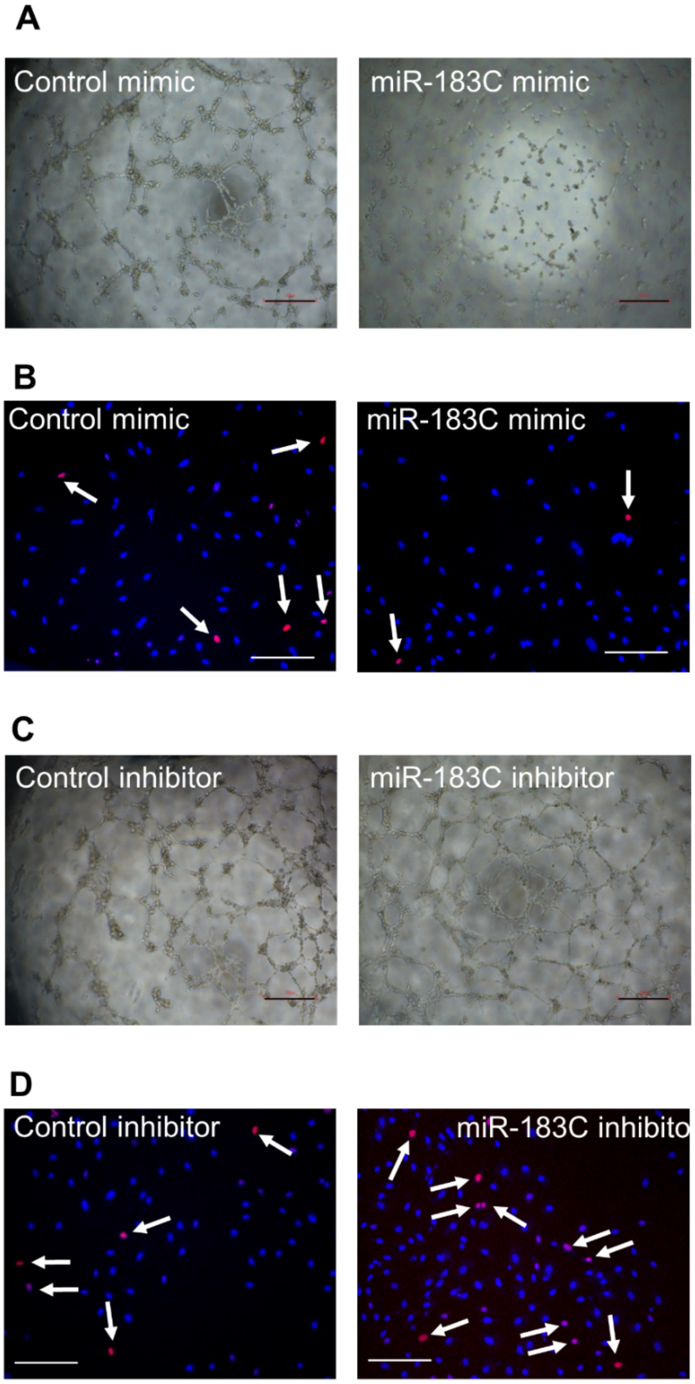

Supplementary Figure 7. Effects of miR-183 cluster inhibition and overexpression in HCECs. (A) Representative microphotographs showing that overexpression of the miR-183 cluster in HCECs reduces the formation of tube-like structures and (B) the number of EdU+ proliferating cells. (C) Representative microphotographs showing that inhibition of the miR-183 cluster in HCECs improves the formation of tube-like structures and (D) increases the number of EdU+ proliferating cells EdU^{+} cells are shown in red and indicated by arrows, DAPI^{+} nuclei in blue. Scale bar = 50μm.

Table 1

	Baseline			14 days	
	**Wild type**	**miR-96-183 KO**		**Wild type**	**miR-96-183 KO**
**Ejection fraction (%)**	63.98±2.47	60.61±9.34		26.27±3.12	22.81±3.06
**Fractional shortening (%)**	*34.55±1.79*	*32.33±5.32*		*14.80±1.41*	*10.61±1.67*
**Cardiac output (ml/min)**	21.86±1.79	16.93±2.97		20.22±2.4	15.40±1.52
**Stroke volume (μl)**	*42.39±3.22*	*34.38±5.8*		*36.56±4.54*	*28.43±2.71*
**ED LV internal diameter (mm)**	3.91±0.15	3.71±0.54		5.50±0.24	5.30±0.21
**ES LV internal diameter (mm)**	*2.57±0.15*	*2.53±0.29*		*4.70±0.23*	*4.77±0.25*
**ED LV chamber volume (μl)**	67.38±6.23	59.61±9.91		140.68±12.05	137.01±11.15
**ES LV chamber volume (μl)**	*24.99±3.51*	*24.36±5.32*		*104.12±10.57*	*108.58±11.60*
**Fibrosis (%)**	-	-		*38.31±6.84*	*35.83±11.73*

Supplementary Table 1. Analysis of cardiac function in Wild type and miR-96-183 KO mice at baseline and after 14 days from MI.
ED: end diastolic; ES: end systolic; LV left ventricle. Data are expressed as mean±SEM. Wild Type n=10; miR-96-183 KO n=13.

Table 2

Age (years)	Gender	Pathology	Surgical procedure
0.8	M	Single ventricle	Glen shunt
1	M	TOF	TOF repair
2	F	Multiple VSD	VSD closure / PA reconstruction
5	M	TOF	Rastelli procedure (RV)
6	F	Bicuspid aortic valve	Ross operation
7	F	Mixed aortic valve disease	Ross operation (RV)
8	M	LVOT /RVOT obstruction	LVOT / RVOT reconstruction
10	M	TOF	Redo PVR
12	F	TOF	Redo PVR
21	F	TOF	Redo PVR
57	M	TOF	Redo PVR
61	M	ASD + mitral valve regurgitation	Mitral valve repair and ASD closure

Supplementary Table 2. Patients characteristics. Age, gender, pathological conditions and surgical procedures performed on human subjects. TOF: Tetralogy of Fallot; VSD: Ventricular Septal Defects; LVOT: Left Ventricular Outflow Tract; RVOT: Right Ventricular Outflow Tract; ASD: Atrial Septal Defect; PA: Pulmonary Artery; PVR: Pulmonary Valve Replacement.
